# The Maryland Medical Journal

**Published:** 1883-07

**Authors:** 


THE MARYLAND MEDICAL JOURNAL.

We see by a card issued by the editors of this sterling periodical that it will hereafter appear as a weekly—being issued every Thursday. Each number will contain sixteen pages double column of solid reading matter. We heartily congratulate our contemporaries on this evidence of the continued prosperity of their journal. May it long flourish to vindicate the truth and dignity of the profession in that noble old Southern commonwealth.

White Paint in Erysipelas.—Mr. Barwell (*Lancet*) has found white paint very efficacious in erysipelas. He reports three cases in which its application was followed by prompt relief of pain and swelling. He mixes carbonate of lead, after the usual manner, with linseed oil, a little turpentine being added as a dryer, and applies freely to the inflamed surface. The remedy probably acts by occlusion of air after the same manner that it does with so much benefit in burns.—*Med. Age, April* 25

Dr. E. E. Montgomery, after operating for lacerated perineum, does not use a catheter, but allows the patient to pass her water, as he does not consider healthy urine disadvantageous for a wound. He also uses compound liquorice powder to keep the stools liquid. He claims good success in both primary and secondary operations.—*Md. Med. Jour., April* 1.

